# Extraction of Clinical Indicators That Are Associated with the Heat/Nonheat and Excess/Deficiency Patterns in Pattern Identifications for Stroke

**DOI:** 10.1155/2015/869894

**Published:** 2015-05-18

**Authors:** Ju Ah Lee, Mi Mi Ko, Jungsup Lee, Byoung-Kab Kang, Terje Alraek, Stephen Birch, Myeong Soo Lee

**Affiliations:** ^1^Medical Research Division, Brain Disease Research Center, Korea Institute of Oriental Medicine, 1672 Yuseongdae-ro, Yuseong-gu, Daejeon 305-811, Republic of Korea; ^2^Korean National Rehabilitation Center, Seoul, Republic of Korea; ^3^University College of Health Sciences, Institute of Acupuncture, 0855 Oslo, Norway; ^4^National Research Center in Complementary and Alternative Medicine (NAFKAM), Department of Community Medicine, Faculty of Health Sciences, UiT The Arctic University of Norway, Tromsø, Norway

## Abstract

The aim of this study is to extract indicators that are associated with the heat/nonheat and excess/deficiency patterns in stroke pattern identification through the large-scale analysis of clinical data. Two experts, who had more than three years of clinical experience with stroke, independently performed the pattern identification. We analyzed indicators of clinical data with two doctors' concurrent diagnoses on the patient's pattern identification. To verify heat/nonheat and excess/deficiency patterns, which are the basic elements of pattern identification, we grouped 960 patients diagnosed as the fire-heat pattern, the Yin deficiency pattern, and the Qi deficiency pattern in to two groups, the heat/nonheat group and the excess/deficiency group. We then extracted significant indicators using univariate and multivariate analysis. As a result of the comparison of 65 indicators, we were able to extract 10 indicators for the heat pattern, 6 for the nonheat pattern, 9 for the excess pattern, and 10 for the deficiency pattern. Extracted indicators in this study can be used for pattern identification in the context of stroke. These are positive indicators from large-scale clinical studies and are greatly expected to be crucial discriminant indicators in individual pattern identification henceforth.

## 1. Introduction

In Korea, many stroke patients receive traditional medical care because the country has its own system of traditional alternative medicine called traditional Korean medicine (TKM), the role of which has been emphasized in stroke management [1]. In TKM and traditional Chinese medicine (TCM), disease is defined as a condition with collapsed equilibrium. TKM doctors make it a rule to restore the imbalance to treat diseases. Pattern identification, a unique diagnostic system of TKM and TCM [2], is the process of overall analysis of clinical data to determine the location, cause, and nature of a patient's disease with an integrative viewpoint that involves the etiology, pathology, and treatment method [3]. There are many classifications in stroke pattern identification, including organ pattern identification, eight-pattern identification, and qi-blood identification. However, the basic elements of pattern identification, such as the discrimination of excessive/deficiency and heat/nonheat patterns, are the same. There were several previous studies on pattern identification, such as a study that diagnosed IBS with the excess/deficiency pattern [4], a study on the period of menstruation [5], and study on traditional Chinese medicine syndromes in women with a frequently recurring cystitis [6]. However, these studies have many limitations because they did not use validated criteria for their TCM diagnoses and hence the reliability of the work. And there are 2 studies that examined reliability of 8-principle diagnosis [7]. However, no study was found to evaluate actual clinical data and extract meaningful indicators. These studies focused only on pattern of disease. Therefore, we adopted an alternative approach, which is to group research subjects into broad categories based on essential patterns of the TCM diagnosis, such as heat/nonheat and excess/deficiency patterns, rather than a detailed TCM diagnosis. First, we made indicators of pattern identification for stroke using Delphi process that is a practical way of generating consensus from a group of expert practitioners [8]. Next, based on the clinical data from a multicenter, large population, the Korean Standard Differentiation of the Symptoms and Signs of Stroke involved 5 categories: the fire-heat pattern, the damp-phlegm pattern, the Yin deficiency pattern, the Qi deficiency pattern, and the blood stasis pattern [9, 10]. Blood stasis pattern was excluded due to rare diagnosis (*n* = 89). Therefore, we extract significant clinical indicators, which affect two pairs of distinct patterns of TCM diagnosis in stroke through data of four patterns: the fire-heat pattern, the damp-phlegm pattern, the Yin deficiency pattern, and the Qi deficiency pattern.

## 2. Participants and Methods

### 2.1. Participants

This study was a community-based multicenter trial. We collected data on stroke patients who had been hospitalized in fifteen oriental medicine university hospitals nationwide from November 2006 to February 2010. Inclusion criteria included having acute stroke with a neurological deficit that persisted for over 24 hours; being finally diagnosed as stroke by imaging, including computerized tomography and magnetic resonance imaging; and agreeing to participate within 30 days of stroke onset. In addition, there were exclusion criteria. Exclusion criteria included traumatic stroke, including epidural hemorrhage and subdural hemorrhage; degenerative brain disease; a stroke concomitant with a brain tumor; and patients unable to communicate symptoms.

### 2.2. Data Collection and Gold Standard for Inclusion

We utilized the case report form (CRF) with a high reliability, collected data on the symptoms and signs, and recorded the score on the 3-point Likert scale for the standardization of stroke diagnosis that had been developed by an expert committee organized by the Korean Institute of Oriental Medicine (KIOM). The principle of symptoms and signs of each patterns extraction was as follows. First, we reflected the characteristic of stroke symptoms to exclude its own symptoms such as hemiparesis, dysphagia, dysarthria, and facial palsy. Second, we identified stroke patterns based on the present state of TKM clinically. Also we considered the association with the previous studies. Finally, we reflected the recent trend of stroke in TKM literature. And two independent physicians conducted this pattern identification. The gold standard was set up as two physicians' concurrent diagnosis which is an identical pattern as a result of diagnostic decision of two TKM physicians. The physicians had at least three years of clinical experiences with stroke and identified the PI of each participating patient. We used data from 960 patients who were diagnosed as the fire-heat pattern, the Yin deficiency pattern, or the Qi deficiency pattern. All of the involved researchers received formal training with standard operation procedures (SOPs) twice a year to exclude all of the possible individual differences.

### 2.3. Assumption for the Extraction of Clinical Indicators of the Heat/Nonheat Pattern and the Excess/Deficiency Pattern

We assumed that the three patterns (the fire-heat pattern, the Yin deficiency pattern, and the Qi deficiency pattern) lie on either side of the space that consists of two axes: the excess/deficiency axis and the heat/nonheat axis. As coldness is mentioned in neither the classical literatures nor the common opinion from the experts, we assumed only the existence and nonexistence of heat. However, this assumption only focused on stroke. We thought normal heat/cold pattern discrimination was replaced by heat/absence of heat signs because cold is not a recognized feature in PI for stroke, while heat is a common pattern and a method is needed to discriminate heat signs.

With the exception of the damp-phlegm pattern and blood stagnation pattern, which are taken as pathological products, we suppose that the fire-heat pattern involves the excess and heat patterns, the Qi deficiency pattern involves deficiency and nonheat patterns, and the Yin deficiency pattern involves deficiency and heat patterns.

### 2.4. Set-Up of an Independent Variable and a Dependent Variable

Among 65 clinical indicators, those with an appearance of less than 5% of the frequency were excluded. Each indicator was established as an independent variable after conversion to the binary form (existence and nonexistence of symptoms or signs). Dependent variables were grouped into two (heat/nonheat and excess/deficiency patterns): the fire-heat pattern and the Yin deficiency pattern to the heat pattern; the fire-heat pattern to the excess pattern; and the Yin deficiency pattern and the Qi deficiency pattern to the deficiency pattern.

### 2.5. Statistical Analysis

A univariate cross tabulation was performed for all of the independent variables and the binary dependent variables. We adopted multilogistic regression analysis for the indicators that showed a significant difference after univariate analysis. The analysis was performed using SAS, version 5.1.

## 3. Results

### 3.1. Number of Participants

The patients included consisted of 444 patients with the fire-heat pattern, 207 with the Yin deficiency pattern, and 313 with the Qi deficiency pattern (Table 1).

### 3.2. Selection of Variables to Be Involved in an Analysis

Six clinical indicators of the 65 indicators on the CRF that appeared at a frequency of less than 5% were found: darkish complexion, headache like flush, headache with nausea, stabbing headache, bluish purple tongue, and purple spots on the tongue. This clinical consideration was generally executed prior to multivariate analysis.

### 3.3. Univariate and Multivariate Analysis to Extract Clinical Indicators for the Heat Pattern and the Nonheat Pattern

After performing a multivariate analysis on the clinical indicators that had a significant difference in univariate analysis, we determined nine important clinical indicators: reddened complexion, blood-shot eyes, aphtha or tongue sore, fetid mouth odor, yellow fur, strong purse, surging purse, heat vexation and aversion to heat, and obesity. These factors displayed odds ratios greater than 1.0. Ten important clinical indicators were as follows: drowsiness, like to lie down, feeling powerless and lazy, pale complexion, pale face and malar flush, pale tongue, teeth-marked tongue, mirror tongue, weak purse, fine purse, and frequent urination. These factors displayed odds ratios of less than 1.0 (Table 2). Whole results of univariate and multivariate analysis to extract clinical indicators for the heat pattern and the nonheat pattern were shown in Supplemental Table 1 available online at http://dx.doi.org/10.1155/2014/869894.

### 3.4. Univariate and Multivariate Analyses to Extract Clinical Indicators in the Excessive Pattern and Deficiency Pattern

After performing a multivariate analysis on clinical indicators that displayed a significant difference on univariate analysis, we identified nine important clinical indicators: pale face and malar flush, reddened complexion, red eye, aphtha or tongue sore, fetid mouth odor, red tongue, dry fur, rapid purse, heat in the palms and soles, and gauntness (odds ratio > 1.0). We identified ten important clinical indicators: looking powerless and lazy, pale complexion, low voice, white fur, teeth-marked tongue, and weak purse (odds ratio < 1.0) (Table 3). Whole results of univariate and multivariate analysis to extract clinical indicators for the excessive pattern and deficiency pattern were shown in Supplemental Table 2.

### 3.5. Comparison of the Clinical Indicators That Explain the Heat/Nonheat Pattern and Excess/Deficiency Pattern

Comparing the results of the heat/nonheat pattern with those of the excess/deficiency pattern, four indicators were found for both heat and excess patterns: reddened complexion, red eye, aphtha or tongue soreness, and fetid mouth odor. Three indicators (pale complexion, teeth-marked tongue, and weak purse) were found in both nonheat and the deficiency patterns (Table 4).

## 4. Discussion

We provide important information for treatment by extracting clinical indicators that have reasonable and objective access to the heat/nonheat pattern and excess/deficiency pattern in acute stroke patients and present the relative risk. There are many diagnostic systems (e.g., organ pattern identification, eight-pattern identification, and qi-blood identification) in TKM [11], but they share certain features. The distinction between excessive/deficiency and heat/nonheat patterns are fundamental to pattern identification. Thus, it is indispensable for the identification of the heat/nonheat pattern and the excess/deficiency pattern. The potential advantages of this approach are the following: it may be easier for physicians to agree on the fundamental pattern types than on complex pattern types; it captures the important and essential factors for stroke pattern identifications; and it can be applied to herbal formulas based on herbal pharmacology. We tried to find clinical data that could identify the heat/nonheat pattern and excess/deficiency pattern by using the Korean Standard Differentiation of the Symptoms and Signs for Stroke that grouped similar patterns into 5 patterns in the context of stroke at TKM. Dissimilar to the previous pattern identification (PI) with 5 patterns agreed upon by stroke experts, we adopted PI with 3 patterns, in which the Damp-phlegm pattern and Blood stagnation pattern were eliminated [12].

To extract indicators that can distinguish heat/nonheat based on this model, we set up Fire-heat/Yin deficiency versus Qi deficiency as independent variables. To extract indicators that could distinguish the excessive/deficiency pattern, Yin deficiency/Qi deficiency and fire-heat patterns were used as independent variables. Clinical data that did not show a significant difference on the chi-square test or that had a total frequency less than 5% were excluded. The others were set as the independent variables in a logistic regression analysis and verified the statistical influence of independent variables on dependent variables.

As a result of the logistic regression analysis, 5 signs and 1 symptom in the excess/deficiency pattern and 8 signs and 6 symptoms in the heat/nonheat pattern were extracted as significant clinical data. This is because the signs like pulse and the tongue indicators were mainly weighted by physicians [13–15]. Physicians diagnose the heat/nonheat pattern and the excess/deficiency pattern by inspecting the existence and nonexistence of these indications. The mere extraction of indicators has certain limitations. It would be beneficial for providing the standardization of TKM in stroke and for evidence-based medicine if we made it possible to assess these factors quantitatively and qualitatively in further studies.

In conclusion, this is the first study aimed to extract objective indicators to differentiate the heat/nonheat pattern and excess/deficiency pattern on the basis of clinical data. This demonstrates how to establish major patterns on a specific disease, how to divide the groups for comparison according to their components, and how to extract significant clinical indicators using a multivariate analysis. Although this study relates to stroke only, this approach has the potential to be applicable to other diseases because it adopts pattern identification, which is an essential diagnostic procedure in TKM treatment.

This study enables us to obtain clinical evidence on pattern diagnosis, which was entirely dependent on the previous literature or expert opinions, and makes it possible to execute evidence-based diagnosis. It is highly likely that we will be able to use more obvious diagnostic evidence from systematic clinical trials in the future as fundamental studies evolve and treatment results accumulate. However, there are some limitations. This study shows only 5 patterns among the various possible patterns of TKM. It does not systematically reflect basic elements in PI from its inception but rather applied a Delphi technique to conventional PI. In this study, we analyzed only the cases that were not assessed to have accompanying patterns. Many physicians, however, understand the possibility of more than 2 patterns during PI and apply it to the clinical field [16, 17]. However, these accompanying patterns are complex. For that reason, this study excluded the accompanying patterns from the analysis and extracted important indicators of each pattern preferentially. Another limitation lies in the variation among raters, which is referred to frequently in TKM research. We made a great effort to minimize error by preparing SOPs and allowed raters to consult more than two SOPs.

## Supplementary Material

Results of univariate analysis in Heat Pattern and Non-heat Pattern & Excessive Pattern and Deficiency Pattern.

## Figures and Tables

**Table 1:** Demographic parameters of enrolled participants.

Characteristics	QD	YD	FH	*P*
*N*	313	207	440	
Sex (M/F)	107/206	91/116	334/106	<0.0001
Age (year)	67.37 ± 11.53	69.27 ± 12.37	65.75 ± 11.98	0.0019
Smoking (none/stop/active)	41/40/232	40/32/135	170/107/163	<0.0001
Drinking (none/stop/active)	68/31/214	58/19/130	214/55/171	<0.0001
Weight (kg)	56.66 ± 9.19	56.97 ± 11.25	64.97 ± 10.78	<0.0001
BMI (kg/m^2^)	22.74 ± 2.99	22.87 ± 4.43	24.16 ± 3.0	<0.0001
Waist circumference (cm)	84.57 ± 8.81	82.77 ± 9.16	88.33 ± 9.34	<0.0001
WHR	0.93 ± 0.11	0.93 ± 0.15	0.95 ± 0.10	NS
TOAST classification				
LAA	51	47	117	0.0073
CE	20	15	34	
SVO	182	95	199	
SOE	7	7	5	
SUE	14	10	22	
Medical history				
TIA (*n*, %)	26 (8.31)	20 (9.66)	35 (7.99)	NS
Hypertension (*n*, %)	180 (57.51)	123 (59.42)	258 (58.77)	NS
Hyperlipidemia (*n*, %)	35 (11.18)	18 (8.70)	54 (12.33)	NS
Diabetes (*n*, %)	82 (26.20)	47 (22.71)	116 (26.48)	NS
Heart disease (*n*, %)	17 (5.43)	12 (5.80)	26 (5.92)	NS

Data was expressed as frequencies for categorical variables and expressed as mean ± standard deviation for continuous variables. YD: Yin deficiency; QD: Qi deficiency; FH: fire-heat; NS: not significant. *P* values were calculated by chi-square test or ANOVA.

**Table 2:** Results of multivariate analysis in excessive pattern and deficiency pattern.

Clinical indicator	Deficiency	Excessive	^c^OR (95% CI)	*P *	^a^OR (95% CI)	*P *
q01_2	46 (8.85)	70 (15.91)	0.615 (0.286, 1.322)	NS	0.721 (0.236, 2.198)	NS
q01_3	146 (28.08)	58 (13.18)	2.346 (1.222, 4.507)	0.0104	2.881 (1.007, 8.243)	0.0485
q02_1	308 (59.23)	133 (30.23)	1.689 (0.94, 3.032)	NS	1.888 (0.713, 4.995)	NS
q02_2	345 (66.35)	125 (28.41)	1.382 (0.762, 2.507)	NS	1.073 (0.386, 2.984)	NS
q03_1_1	179 (34.42)	22 (5)	2.947 (1.178, 7.375)	0.0209	2.826 (0.622, 12.835)	NS
q03_1_2	123 (23.65)	64 (14.55)	1.179 (0.537, 2.587)	NS	0.754 (0.23, 2.467)	NS
q03_1_3	14 (2.69)	3 (0.68)	2.772 (0.42, 18.321)	NS	0.759 (0.055, 10.523)	NS
q03_1_4	90 (17.31)	32 (7.27)	2.646 (1.09, 6.425)	0.0315	2.302 (0.58, 9.135)	NS
q03_1_5	60 (11.54)	284 (64.55)	0.2 (0.094, 0.424)	<0.0001	0.103 (0.03, 0.353)	0.0003
q03_2	79 (15.19)	39 (8.86)	1.33 (0.622, 2.842)	NS	0.461 (0.142, 1.493)	NS
q04_3_2	15 (2.88)	31 (7.05)	0.139 (0.046, 0.42)	0.0005	0.157 (0.023, 1.05)	NS
q05_1	275 (52.88)	190 (43.18)	1.279 (0.78, 2.099)	NS	1.169 (0.538, 2.54)	NS
q06_1	74 (14.23)	90 (20.45)	0.859 (0.466, 1.585)	NS	2.504 (0.899, 6.973)	NS
q06_2	207 (39.81)	71 (16.14)	1.623 (0.858, 3.07)	NS	1.552 (0.505, 4.774)	NS
q07_1_1	23 (4.42)	37 (8.41)	0.306 (0.119, 0.79)	0.0144	0.133 (0.03, 0.584)	0.0075
q07_1_2	59 (11.35)	111 (25.23)	0.439 (0.228, 0.844)	0.0135	0.331 (0.122, 0.9)	0.0304
q07_4	157 (30.19)	182 (41.36)	0.515 (0.314, 0.842)	0.0082	0.679 (0.323, 1.429)	NS
q08_1_1	138 (26.54)	38 (8.64)	3.625 (1.229, 10.692)	0.0196	4.806 (0.962, 24.009)	NS
q08_1_2	221 (42.5)	147 (33.41)	2.079 (0.806, 5.364)	NS	1.703 (0.402, 7.222)	NS
q08_1_3	140 (26.92)	224 (50.91)	0.81 (0.319, 2.054)	NS	0.626 (0.149, 2.626)	NS
q08_2_1	101 (19.42)	216 (49.09)	0.281 (0.14, 0.563)	0.0003	0.487 (0.159, 1.488)	NS
q08_2_2	244 (46.92)	136 (30.91)	0.809 (0.437, 1.498)	NS	1.466 (0.553, 3.883)	NS
q08_3_1	102 (19.62)	201 (45.68)	0.56 (0.32, 0.98)	0.0422	0.439 (0.188, 1.026)	NS
q08_4_1	102 (19.62)	110 (25)	2.073 (1.178, 3.647)	0.0114	2.011 (0.841, 4.808)	NS
q08_5_1	85 (16.35)	40 (9.09)	2.317 (1.122, 4.784)	0.0231	4.518 (1.616, 12.632)	0.004
q08_5_4	34 (6.54)	15 (3.41)	4.956 (1.61, 15.257)	0.0053	15.977 (1.97, 129.6)	0.0095
q10_1	128 (24.62)	150 (34.09)	0.657 (0.389, 1.108)	NS	1.572 (0.672, 3.679)	NS
q11_1	205 (39.42)	146 (33.18)	1.799 (1.1, 2.941)	0.0193	2.598 (1.166, 5.788)	0.0195
q11_3	115 (22.12)	125 (28.41)	0.927 (0.543, 1.581)	NS	0.92 (0.412, 2.052)	NS
q12_4	73 (14.04)	86 (19.55)	0.683 (0.369, 1.262)	NS	1.262 (0.463, 3.446)	NS
q13_1	47 (9.04)	18 (4.09)	1.403 (0.532, 3.695)	NS	2.322 (0.443, 12.154)	NS
q14_1_1	116 (22.31)	189 (42.95)	0.957 (0.517, 1.772)	NS	0.398 (0.148, 1.068)	NS
q14_1_2	232 (44.62)	94 (21.36)	1.726 (0.934, 3.189)	NS	1.318 (0.508, 3.419)	NS
q14_2_1	127 (24.42)	38 (8.64)	1.097 (0.525, 2.294)	NS	1.619 (0.504, 5.204)	NS
q14_2_2	137 (26.35)	199 (45.23)	0.637 (0.371, 1.095)	NS	0.668 (0.285, 1.564)	NS
q14_3_1	108 (20.77)	297 (67.5)	0.494 (0.271, 0.9)	0.0212	0.441 (0.176, 1.104)	NS
q14_3_2	298 (57.31)	55 (12.5)	2.026 (1.037, 3.957)	0.0388	2.024 (0.726, 5.642)	NS
q14_4	229 (44.04)	23 (5.23)	6.098 (3.055, 12.172)	<0.0001	3.416 (1.208, 9.659)	0.0205
q14_5	90 (17.31)	161 (36.59)	0.912 (0.544, 1.528)	NS	0.755 (0.334, 1.71)	NS
q14_7	13 (2.5)	96 (21.82)	0.093 (0.034, 0.254)	<0.0001	0.087 (0.02, 0.38)	0.0012
q15_1	208 (40)	292 (66.36)	0.503 (0.308, 0.821)	0.006	0.341 (0.156, 0.744)	0.0069
q15_2_1	49 (9.42)	74 (16.82)	1.411 (0.68, 2.927)	NS	0.782 (0.272, 2.249)	NS
q15_2_2	35 (6.73)	69 (15.68)	0.423 (0.192, 0.931)	0.0327	0.853 (0.256, 2.848)	NS
q15_2_3	85 (16.35)	29 (6.59)	2.074 (0.959, 4.484)	NS	2.55 (0.781, 8.33)	NS
q16_1_1	76 (14.62)	125 (28.41)	0.44 (0.246, 0.786)	0.0056	0.391 (0.138, 1.113)	NS

Data was expressed as yes (%). Excessive: fire-heat pattern; deficiency: Qi deficiency pattern and Yin deficiency Pattern; ^c^OR means crude odds ratio and ^a^OR means odds ratio adjusted by sex, age, BMI, WHR, smoking, and drinking status. NS: not significant.

**Table 3:** Results of multivariate analysis in heat pattern and nonheat pattern.

Clinical indicator	Heat	Nonheat	^c^OR (95% CI)	*P*	^a^OR (95% CI)	*P*
q01_2	97 (14.99)	19 (6.07)	2.891 (1.119, 7.464)	0.0283	1.471 (0.369, 5.868)	NS
q01_3	103 (15.92)	101 (32.27)	0.74 (0.416, 1.319)	NS	0.63 (0.259, 1.532)	NS
q02_1	233 (36.01)	208 (66.45)	0.729 (0.397, 1.337)	NS	0.579 (0.235, 1.43)	NS
q02_2	229 (35.39)	241 (77)	0.409 (0.219, 0.764)	0.005	0.516 (0.213, 1.249)	NS
q03_1_1	43 (6.65)	158 (50.48)	0.205 (0.112, 0.375)	<0.0001	0.279 (0.112, 0.694)	0.006
q03_1_4	105 (16.23)	17 (5.43)	5.985 (2.764, 12.961)	<0.0001	4.776 (1.591, 14.335)	0.0053
q03_1_5	321 (49.61)	23 (7.35)	4.581 (2.402, 8.735)	<0.0001	6.929 (2.589, 18.547)	0.0001
q03_2	69 (10.66)	49 (15.65)	0.801 (0.419, 1.531)	NS	1.025 (0.372, 2.824)	NS
q04_3_2	40 (6.18)	6 (1.92)	2.034 (0.487, 8.495)	NS	1.854 (0.267, 12.87)	NS
q05_1	288 (44.51)	177 (56.55)	0.916 (0.52, 1.614)	NS	1.022 (0.436, 2.394)	NS
q06_1	130 (20.09)	34 (10.86)	1.191 (0.618, 2.292)	NS	0.946 (0.316, 2.83)	NS
q06_2	131 (20.25)	147 (46.96)	0.496 (0.279, 0.88)	0.0165	0.361 (0.141, 0.921)	0.0329
q07_1_1	48 (7.42)	12 (3.83)	2.203 (0.796, 6.096)	NS	2.863 (0.572, 14.328)	NS
q07_1_2	138 (21.33)	32 (10.22)	2.727 (1.324, 5.614)	0.0065	2.713 (0.917, 8.023)	NS
q07_4	250 (38.64)	89 (28.43)	0.958 (0.583, 1.576)	NS	0.794 (0.388, 1.624)	NS
q08_1_1	62 (9.58)	114 (36.42)	0.425 (0.145, 1.25)	NS	0.974 (0.197, 4.817)	NS
q08_1_2	228 (35.24)	140 (44.73)	0.657 (0.24, 1.8)	NS	1.516 (0.337, 6.814)	NS
q08_1_3	318 (49.15)	46 (14.7)	3.119 (1.102, 8.826)	0.0321	9.338 (1.918, 45.477)	0.0057
q08_2_1	269 (41.58)	48 (15.34)	2.023 (0.987, 4.148)	NS	2.688 (0.952, 7.588)	NS
q08_2_2	205 (31.68)	175 (55.91)	0.436 (0.239, 0.795)	0.0067	0.478 (0.202, 1.132)	NS
q08_3_1	235 (36.32)	68 (21.73)	0.812 (0.452, 1.461)	NS	0.633 (0.27, 1.48)	NS
q08_4_1	178 (27.51)	34 (10.86)	2.175 (1.151, 4.111)	0.0167	4.771 (1.71, 13.318)	0.0028
q08_5_1	61 (9.43)	64 (20.45)	0.46 (0.242, 0.877)	0.0182	0.264 (0.106, 0.662)	0.0045
q08_5_4	41 (6.34)	8 (2.56)	3.018 (0.902, 10.102)	NS	6.052 (1.018, 35.979)	0.0478
q09_1	131 (20.25)	44 (14.06)	1.829 (0.715, 4.68)	NS	1.151 (0.303, 4.368)	NS
q09_3	99 (15.3)	26 (8.31)	0.81 (0.254, 2.575)	NS	1.356 (0.277, 6.639)	NS
q10_2	140 (21.64)	45 (14.38)	1.164 (0.632, 2.143)	NS	1.455 (0.571, 3.709)	NS
q11_3	184 (28.44)	56 (17.89)	1.199 (0.679, 2.12)	NS	1.356 (0.607, 3.031)	NS
q14_1_1	241 (37.25)	64 (20.45)	0.971 (0.502, 1.876)	NS	1.336 (0.503, 3.551)	NS
q14_1_2	162 (25.04)	164 (52.4)	0.472 (0.26, 0.855)	0.0133	0.491 (0.202, 1.191)	NS
q14_2_1	70 (10.82)	95 (30.35)	1.376 (0.704, 2.691)	NS	1.619 (0.582, 4.504)	NS
q14_2_2	285 (44.05)	51 (16.29)	3.207 (1.763, 5.833)	0.0001	3.874 (1.625, 9.235)	0.0022
q14_3_1	350 (54.1)	55 (17.57)	1.34 (0.679, 2.644)	NS	1.049 (0.387, 2.846)	NS
q14_3_2	149 (23.03)	204 (65.18)	0.434 (0.231, 0.816)	0.0096	0.365 (0.144, 0.921)	0.0328
q14_4	114 (17.62)	138 (44.09)	0.933 (0.532, 1.636)	NS	2.03 (0.847, 4.86)	NS
q14_5	201 (31.07)	50 (15.97)	1.503 (0.82, 2.755)	NS	2.104 (0.887, 4.993)	NS
q14_7	101 (15.61)	8 (2.56)	2.97 (1.003, 8.795)	0.0495	2.55 (0.554, 11.732)	NS
q15_1	390 (60.28)	110 (35.14)	1.446 (0.885, 2.364)	NS	1.517 (0.7, 3.112)	NS
q15_2_1	107 (16.54)	16 (5.11)	4.983 (1.857, 13.37)	0.0014	6.95 (1.646, 29.345)	0.0083
q15_2_2	87 (13.45)	17 (5.43)	0.988 (0.417, 2.341)	NS	0.502 (0.146, 1.727)	NS
q15_2_3	54 (8.35)	60 (19.17)	0.51 (0.253, 1.027)	NS	0.569 (0.204, 1.586)	NS
q15_3	85 (13.14)	22 (7.03)	1.61 (0.675, 3.84)	NS	1.383 (0.384, 4.986)	NS
q16_1_1	161 (24.88)	40 (12.78)	1.597 (0.799, 3.191)	NS	1.877 (0.626, 5.63)	NS
q16_1_2	140 (21.64)	47 (15.02)	1.731 (0.83, 3.61)	NS	1.06 (0.362, 3.102)	NS

Data was expressed as yes (%). Heat: fire-heat pattern and Yin deficiency pattern; Nonheat: Qi deficiency pattern; ^c^OR means crude odds ratio and ^a^OR means odds ratio adjusted by sex, age, BMI, WHR, smoking, and drinking status. NS: not significant.

**Table 4:** Comparison of clinical indicators that explain heat/nonheat pattern and excess/deficiency.

	Excess	Deficiency
Heat	Reddened complexion Red eye Aphtha or tongue-sore Fetid mouth odor	

Nonheat		Pale complexion, Teeth-marked tongue Weak purse
