# Supplementary material for: Extraction of Clinical Indicators That Are Associated with the Heat/Nonheat and Excess/Deficiency Patterns in Pattern Identifications for Stroke
Source: Evid Based Complement Alternat Med. 2015 May 18;2015:869894. doi: 10.1155/2015/869894 (PMC4452190; doi:10.1155/2015/869894)
Supplement: Supplementary file 1 — Results of univariate analysis in Heat Pattern and Non-heat Pattern & Excessive Pattern and Deficiency Pattern. [file 869894.f1.docx]

**Supplement 1.** Results of univariate analysis in Heat Pattern and Non-heat Pattern

| Clinical indicator | Heat | Non-heat | OR (95% CI) | *p* |
| --- | --- | --- | --- | --- |
| q01_1 | 444 (68.62) | 230 (73.48) | 0.789 (0.584, 1.066) | 0.1229 |
| q01_2 | 97 (14.99) | 19 (6.07) | 2.729 (1.636, 4.552) | <.0001 |
| q01_3 | 103 (15.92) | 101 (32.27) | 0.397 (0.289, 0.546) | <.0001 |
| q02_1 | 233 (36.01) | 208 (66.45) | 0.284 (0.214, 0.378) | <.0001 |
| q02_2 | 229 (35.39) | 241 (77) | 0.164 (0.120, 0.223) | <.0001 |
| q03_1_1 | 43 (6.65) | 158 (50.48) | 0.070 (0.048, 0.102) | <.0001 |
| q03_1_2 | 118 (18.24) | 69 (22.04) | 0.789 (0.565,1.101) | 0.1627 |
| q03_1_3 | 9 (1.39) | 8 (2.56) | 0.538 (0.206, 1.408) | 0.1996 |
| q03_1_4 | 105 (16.23) | 17 (5.43) | 3.373 (1.982, 5.74) | <.0001 |
| q03_1_5 | 321 (49.61) | 23 (7.35) | 12.415 (7.903, 19.504) | <.0001 |
| q03_2 | 69 (10.66) | 49 (15.65) | 0.643 (0.434, 0.954) | 0.0273 |
| q03_3 | 94 (14.53) | 44 (14.06) | 1.039 (0.706, 1.530) | 0.8454 |
| q03_4 | 98 (15.15) | 39 (12.46) | 1.254 (0.842,1.868) | 0.2646 |
| q04_1 | 156 (24.11) | 80 (25.56) | 0.925 (0.678, 1.263) | 0.6253 |
| q04_2 | 97 (14.99) | 46 (14.7) | 1.024 (0.7, 1.497) | 0.904 |
| q04_3_1 | 117 (18.08) | 53 (16.93) | 1.083 (0.758, 1.547) | 0.6616 |
| q04_3_2 | 40 (6.18) | 6 (1.92) | 3.372 (1.414, 8.040) | 0.0037 |
| q04_3_3 | 29 (4.48) | 20 (6.39) | 0.688 (0.383, 1.236) | 0.2081 |
| q04_3_4 | 15 (2.32) | 6 (1.92) | 1.214 (0.467, 3.161) | 0.6902 |
| q04_4 | 72 (11.13) | 34 (10.86) | 1.028 (0.667, 1.583) | 0.902 |
| q05_1 | 288 (44.51) | 177 (56.55) | 0.616 (0.470, 0.809) | 0.0005 |
| q05_2 | 182 (28.13) | 84 (26.84) | 1.067 (0.788, 1.445) | 0.6748 |
| q06_1 | 130 (20.09) | 34 (10.86) | 2.063 (1.377, 3.093) | 0.0004 |
| q06_2 | 131 (20.25) | 147 (46.96) | 0.287 (0.214, 0.384) | <.0001 |
| q07_1_1 | 48 (7.42) | 12 (3.83) | 2.01 (1.052, 3.841) | 0.0315 |
| q07_1_2 | 138 (21.33) | 32 (10.22) | 2.381 (1.578, 3.592) | <.0001 |
| q07_2 | 344 (53.17) | 146 (46.65) | 1.299 (0.991, 1.702) | 0.0581 |
| q07_3 | 178 (27.51) | 84 (26.84) | 1.035 (0.764, 1.402) | 0.8259 |
| q07_4 | 250 (38.64) | 89 (28.43) | 1.585 (1.183, 2.123) | 0.0019 |
| q08_1_1 | 62 (9.58) | 114 (36.42) | 0.185 (0.131, 0.262) | <.0001 |
| q08_1_2 | 228 (35.24) | 140 (44.73) | 0.672 (0.511, 0.885) | 0.0046 |
| q08_1_3 | 318 (49.15) | 46 (14.7) | 5.610 (3.958, 7.952) | <.0001 |
| q08_1_4 | 22 (3.4) | 6 (1.92) | 1.801 (0.723, 4.488) | 0.2004 |
| q08_2_1 | 269 (41.58) | 48 (15.34) | 3.929 (2.783, 5.547) | <.0001 |
| q08_2_2 | 205 (31.68) | 175 (55.91) | 0.366 (0.277, 0.483) | <.0001 |
| q08_3_1 | 235 (36.32) | 68 (21.73) | 2.055 (1.503, 2.810) | <.0001 |
| q08_4_1 | 178 (27.51) | 34 (10.86) | 3.114 (2.097, 4.626) | <.0001 |
| q08_5_1 | 61 (9.43) | 64 (20.45) | 0.405 (0.277, 0.593) | <.0001 |
| q08_5_2 | 77 (11.9) | 48 (15.34) | 0.746 (0.506, 1.100) | 0.1383 |
| q08_5_3 | 5 (0.77) | 1 (0.32) | 2.430 (0.288, 20.887) | 0.4035 |
| q08_5_4 | 41 (6.34) | 8 (2.56) | 2.579 (1.194, 5.571) | 0.0126 |
| q09_1 | 131 (20.25) | 44 (14.06) | 1.552 (1.07, 2.251) | 0.0199 |
| q09_2 | 16 (2.47) | 7 (2.24) | 1.108 (0.451, 2.723) | 0.8222 |
| q09_3 | 99 (15.3) | 26 (8.31) | 1.994 (1.265, 3.143) | 0.0025 |
| q09_4 | 56 (8.66) | 25 (7.99) | 1.092 (0.667, 1.785) | 0.727 |
| q10_1 | 193 (29.83) | 85 (27.16) | 1.140 (0.844, 1.540) | 0.392 |
| q10_2 | 140 (21.64) | 45 (14.38) | 1.645 (1.139, 2.374) | 0.0075 |
| q11_1 | 229 (35.39) | 122 (38.98) | 0.858 (0.649, 1.133) | 0.2798 |
| q11_3 | 184 (28.44) | 56 (17.89) | 1.824 (1.304, 2.552) | 0.0004 |
| q12_2 | 204 (31.53) | 97 (30.99) | 1.025 (0.766, 1.372) | 0.8658 |
| q12_3 | 178 (27.51) | 73 (23.32) | 1.248 (0.912, 1.708) | 0.1662 |
| q12_4 | 114 (17.62) | 45 (14.38) | 1.274 (0.876, 1.853) | 0.2052 |
| q13_1 | 39 (6.03) | 26 (8.31) | 0.708 (0.423, 1.186 ) | 0.1877 |
| q13_2 | 65 (10.05) | 31 (9.9) | 1.016 (0.647, 1.595) | 0.9451 |
| q14_1_1 | 241 (37.25) | 64 (20.45) | 2.310 (1.681, 3.173) | <.0001 |
| q14_1_2 | 162 (25.04) | 164 (52.4) | 0.304 (0.228, 0.403) | <.0001 |
| q14_2_1 | 70 (10.82) | 95 (30.35) | 0.278 (0.197, 0.393) | <.0001 |
| q14_2_2 | 285 (44.05) | 51 (16.29) | 4.045 (2.885, 5.670) | <.0001 |
| q14_3_1 | 350 (54.1) | 55 (17.57) | 5.528 (3.976, 7.686) | <.0001 |
| q14_3_2 | 149 (23.03) | 204 (65.18) | 0.160 (0.119, 0.215) | <.0001 |
| q14_4 | 114 (17.62) | 138 (44.09) | 0.271 (0.201, 0.367) | <.0001 |
| q14_5 | 201 (31.07) | 50 (15.97) | 2.371 (1.679, 3.348) | <.0001 |
| q14_6 | 35 (5.41) | 24 (7.67) | 0.689 (0.402, 1.179) | 0.1721 |
| q14_7 | 101 (15.61) | 8 (2.56) | 7.052 (3.387, 14.684) | <.0001 |
| q15_1 | 390 (60.28) | 110 (35.14) | 2.801 (2.116, 3.707) | <.0001 |
| q15_2_1 | 107 (16.54) | 16 (5.11) | 3.678 (2.135, 6.338) | <.0001 |
| q15_2_2 | 87 (13.45) | 17 (5.43) | 2.705 (1.579, 4.635) | 0.0002 |
| q15_2_3 | 54 (8.35) | 60 (19.17) | 0.384 (0.258, 0.571) | <.0001 |
| q15_3 | 85 (13.14) | 22 (7.03) | 2.001 (1.226, 3.265) | 0.0048 |
| q16_1_1 | 161 (24.88) | 40 (12.78) | 2.261 (1.551, 3.295) | <.0001 |
| q16_1_2 | 140 (21.64) | 47 (15.02) | 1.563 (1.088, 2.245) | 0.0152 |

Data was expressed as yes (%).Heat: Fire-Heat Pattern and Yin Deficiency Pattern; Non-heat: Qi Deficiency Pattern;

**Supplement 2.** Results of univariate analysis in Excessive Pattern and Deficiency Pattern

| Clinical indicator | Deficiency | Excessive | OR (95% CI) | *p* |
| --- | --- | --- | --- | --- |
| q01_1 | 373 (71.73) | 301 (68.41) | 1.172 (0.888, 1.546) | 0.2622 |
| q01_2 | 46 (8.85) | 70 (15.91) | 0.513 (0.345, 0.762) | 0.0008 |
| q01_3 | 146 (28.08) | 58 (13.18) | 2.571 (1.837, 3.598) | <.0001 |
| q02_1 | 308 (59.23) | 133 (30.23) | 3.354 (2.564, 4.386) | <.0001 |
| q02_2 | 345 (66.35) | 125 (28.41) | 4.968 (3.771, 6.545) | <.0001 |
| q03_1_1 | 179 (34.42) | 22 (5) | 9.974 (6.263, 15.883) | <.0001 |
| q03_1_2 | 123 (23.65) | 64 (14.55) | 1.820 (1.304, 2.541) | 0.0004 |
| q03_1_3 | 14 (2.69) | 3 (0.68) | 4.030 (1.151, 14.116) | 0.0186 |
| q03_1_4 | 90 (17.31) | 32 (7.27) | 2.669 (1.744, 4.084) | <.0001 |
| q03_1_5 | 60 (11.54) | 284 (64.55) | 0.072 (0.051, 0.0999) | <.0001 |
| q03_2 | 79 (15.19) | 39 (8.86) | 1.842 (1.226, 2.766) | 0.0029 |
| q03_3 | 77 (14.81) | 61 (13.86) | 1.080 (0.751, 1.552) | 0.6778 |
| q03_4 | 82 (15.77) | 55 (12.5) | 1.311 (0.907, 1.894) | 0.149 |
| q04_1 | 128 (24.62) | 108 (24.55) | 1.004 (0.747, 1.348) | 0.98 |
| q04_2 | 72 (13.85) | 71 (16.14) | 0.835 (0.585, 1.192) | 0.3207 |
| q04_3_1 | 92 (17.69) | 78 (17.73) | 0.998 (0.715, 1.391) | 0.9887 |
| q04_3_2 | 15 (2.88) | 31 (7.05) | 0.392 (0.209, 0.736) | 0.0026 |
| q04_3_3 | 25 (4.81) | 24 (5.45) | 0.875 (0.493, 1.556) | 0.65 |
| q04_3_4 | 12 (2.31) | 9 (2.05) | 1.131 (0.472, 2.710) | 0.782 |
| q04_4 | 63 (12.12) | 43 (9.77) | 1.273 (0.844, 1.919) | 0.2485 |
| q05_1 | 275 (52.88) | 190 (43.18) | 1.477 (1.144, 1.907) | 0.0027 |
| q05_2 | 154 (29.62) | 112 (25.45) | 1.232 (0.926, 1.639) | 0.1512 |
| q06_1 | 74 (14.23) | 90 (20.45) | 0.645 (0.460, 0.905) | 0.0107 |
| q06_2 | 207 (39.81) | 71 (16.14) | 3.437 (2.524,4.681) | <.0001 |
| q07_1_1 | 23 (4.42) | 37 (8.41) | 0.504 (0.295, 0.862) | 0.011 |
| q07_1_2 | 59 (11.35) | 111 (25.23) | 0.379 (0.268, 0.536) | <.0001 |
| q07_2 | 267 (51.35) | 223 (50.68) | 1.0269 (0.797, 1.324) | 0.8374 |
| q07_3 | 140 (26.92) | 122 (27.73) | 0.960 (0.722, 1.277) | 0.7805 |
| q07_4 | 157 (30.19) | 182 (41.36) | 0.613 (0.470, 0.800) | 0.0003 |
| q08_1_1 | 138 (26.54) | 38 (8.64) | 3.822 (2.599, 5.619) | <.0001 |
| q08_1_2 | 221 (42.5) | 147 (33.41) | 1.473 (1.132, 1.918) | 0.0039 |
| q08_1_3 | 140 (26.92) | 224 (50.91) | 0.355 (0.271, 0.465) | <.0001 |
| q08_1_4 | 11 (2.12) | 17 (3.86) | 0.538 (0.2492, 1.161) | 0.1087 |
| q08_2_1 | 101 (19.42) | 216 (49.09) | 0.25 (0.188, 0.333) | <.0001 |
| q08_2_2 | 244 (46.92) | 136 (30.91) | 1.976 (1.515, 2.577) | <.0001 |
| q08_3_1 | 102 (19.62) | 201 (45.68) | 0.290 (0.218, 0.386) | <.0001 |
| q08_4_1 | 102 (19.62) | 110 (25) | 0.732 (0.539, 0.994) | 0.0451 |
| q08_5_1 | 85 (16.35) | 40 (9.09) | 1.954(1.310, 2.914) | 0.0009 |
| q08_5_2 | 74 (14.23) | 51 (11.59) | 1.266 (0.864,1.854) | 0.2259 |
| q08_5_3 | 1 (0.19) | 5 (1.14) | 0.168 (0.020, 1.440) | 0.0644 |
| q08_5_4 | 34 (6.54) | 15 (3.41) | 1.982 (1.065, 3.690) | 0.0282 |
| q09_1 | 91 (17.5) | 84 (19.09) | 0.899(0.648, 1.248) | 0.5247 |
| q09_2 | 12 (2.31) | 11 (2.5) | 0.921 (0.403, 2.109) | 0.8461 |
| q09_3 | 59 (11.35) | 66 (15) | 0.725 (0.498, 1.057) | 0.0937 |
| q09_4 | 50 (9.62) | 31 (7.05) | 1.404 (0.880, 2.240) | 0.1535 |
| q10_1 | 128 (24.62) | 150 (34.09) | 0.631 (0.477, 0.836) | 0.0013 |
| q10_2 | 94 (18.08) | 91 (20.68) | 0.846 (0.614, 1.167) | 0.3079 |
| q11_1 | 205 (39.42) | 146 (33.18) | 1.311 (1.005, 1.708) | 0.0454 |
| q11_3 | 115 (22.12) | 125 (28.41) | 0.716 (0.534, 0.959) | 0.0248 |
| q12_2 | 163 (31.35) | 138 (31.36) | 0.999 (0.76, 1.314) | 0.9954 |
| q12_3 | 129 (24.81) | 122 (27.73) | 0.86 (0.645, 1.147) | 0.305 |
| q12_4 | 73 (14.04) | 86 (19.55) | 0.672 (0.478, 0.946) | 0.0222 |
| q13_1 | 47 (9.04) | 18 (4.09) | 2.330 (1.332, 4.074) | 0.0024 |
| q13_2 | 48 (9.23) | 48 (10.91) | 0.831 (0.545, 1.266) | 0.3878 |
| q14_1_1 | 116 (22.31) | 189 (42.95) | 0.381 (0.288, 0.504) | <.0001 |
| q14_1_2 | 232 (44.62) | 94 (21.36) | 2.965 (2.227, 3.947) | <.0001 |
| q14_2_1 | 127 (24.42) | 38 (8.64) | 3.419 (2.319, 5.04) | <.0001 |
| q14_2_2 | 137 (26.35) | 199 (45.23) | 0.433 (0.330, 0.568) | <.0001 |
| q14_3_1 | 108 (20.77) | 297 (67.5) | 0.126 (0.094, 0.169) | <.0001 |
| q14_3_2 | 298 (57.31) | 55 (12.5) | 9.396 (6.744, 13.092) | <.0001 |
| q14_4 | 229 (44.04) | 23 (5.23) | 14.268 (9.060, 22.468) | <.0001 |
| q14_5 | 90 (17.31) | 161 (36.59) | 0.363 (0.269, 0.489) | <.0001 |
| q14_6 | 38 (7.31) | 21 (4.77) | 1.573 (0.908, 2.723) | 0.1032 |
| q14_7 | 13 (2.5) | 96 (21.82) | 0.092 (0.051, 0.167) | <.0001 |
| q15_1 | 208 (40) | 292 (66.36) | 0.338 (0.259, 0.440) | <.0001 |
| q15_2_1 | 49 (9.42) | 74 (16.82) | 0.515 (0.350, 0.757) | 0.0006 |
| q15_2_2 | 35 (6.73) | 69 (15.68) | 0.388 (0.253, 0.596) | <.0001 |
| q15_2_3 | 85 (16.35) | 29 (6.59) | 2.769 (1.779, 4.311) | <.0001 |
| q15_3 | 59 (11.35) | 48 (10.91) | 1.045 (0.698, 1.566) | 0.8302 |
| q16_1_1 | 76 (14.62) | 125 (28.41) | 0.431 (0.313, 0.594) | <.0001 |
| q16_1_2 | 100 (19.23) | 87 (19.77) | 0.966 (0.701, 1.331) | 0.8327 |

Data was expressed as yes (%).Excessive: Fire-Heat Pattern; Deficiency: Qi Deficiency Pattern and Yin Deficiency Pattern
